# Improvement of volatile aromatic compound levels and sensory quality of distilled *soju* derived from *Saccharomyces cerevisiae* and *Wickerhamomyces anomalus* co-fermentation

**DOI:** 10.1016/j.fochx.2024.101368

**Published:** 2024-04-12

**Authors:** Kyu-Taek Choi, Su-Hyun Lee, Yeong-Jun Kim, Jun-Su Choi, Sae-Byuk Lee

**Affiliations:** a School of Food Science and Biotechnology, Kyungpook National University, Daegu 41566, South Korea; b Institute of Fermentation Biotechnology, Kyungpook National University, Daegu 41566, South Korea

**Keywords:** Distilled *soju*, *Wickerhamomyces anomalus*, Volatile aromatic compounds, Co-fermentation

## Abstract

Distilled *soju*, a Korean traditional alcoholic beverage, is produced by fermenting rice with a variety of microorganisms including molds, yeasts, and lactic acid-producing bacteria, followed by distillation. Our study sought to improve the quality of distilled *soju* through co-fermentation with *Saccharomyces cerevisiae* and *Wickerhamomyces anomalus*, known for producing volatile aromatic compounds during the early stages of fermentation. Analysis of volatile aromatic compounds in co-fermented distilled *soju* revealed a substantial increase in compounds with boiling points below 200 °C. Among them, ethyl hexanoate, isobutanol, and isoamyl alcohol were identified as the major volatile aromatic compounds based on Log2 fold change analyses of the volatile aromatic compound pattern. In sensory evaluation, co-fermented distilled *soju* received higher scores in terms of odor and overall preference. Therefore, incorporating *W. anomalus* may improve the quality of distilled *soju*.

## Highlights

• Co-fermentation with *W. anomalus* enhances volatile aromatic compound levels.
• Distillation concentrates volatile aromatic compounds with boiling points < 200 °C.
• Ethyl hexanoate, isobutanol, and isoamyl alcohol are major volatiles in distilled *soju*.
• Co-fermentation with *W. anomalus* improves the sensory quality of distilled *soju*.

## 1 Introduction

Distilled *soju*, a traditional Korean alcoholic beverage, is made by distilling the fermentation products of grains such as rice, known as *takju*. The fermentation process is influenced by diverse microorganisms, including fungi, yeast, and lactic acid-producing bacteria, all of which impart unique characteristics to the end product. Therefore, co-fermented distilled *soju* has a more complex and rich aroma compared with diluted *soju*, which is typically mixed with a continuously distilled spirit containing 95% (*v*/v) ethanol and added sweetener (Hong et al., 2020; Kim, 2022; Kim & Lee, 2022). Throughout the fermentation process, various compounds such as sugars, organic acids, amino acids, and volatile aromatic compounds are produced from the raw materials via the metabolic processes of the microbial community (Choi et al., 2023; Kang et al., 2015). Moreover, different brewing and distillation methods can substantially influence the quality of distilled *soju*. Typically, harmful substances with lower boiling points than water such as methanol and acetaldehyde (i.e., the heads) are initially discarded, after which the subsequent products (i.e., the hearts) are collected. Substances such as furfural and fusel alcohols (also known as fusel oils), which exhibit higher boiling points than water, can detrimentally affect the spirit's quality and are naturally eliminated in the tails (Lee et al., 2017). Numerous studies have been conducted in Korea to enhance the quality of distilled *soju*, including studies on the fermentation of *takju* (also known as makgeolli) (Choi et al., 2013; Choi et al., 2015; Choi et al., 2017; Lee et al., 2015), distillation methodologies applied to makgeolli (Lee et al., 2017), and the post-distillation aging phase (Kim & Lee, 2019).

Recent trends in alcoholic beverage research have sparked interest in non-*Saccharomyces* yeasts, which are known for producing diverse aromatic compounds such as esters, aldehydes, higher alcohols, and terpenes during the early stages of fermentation (Borren & Tian, 2020; Ellis et al., 2022; Tufariello et al., 2021). Due to their low alcohol tolerance, non-*Saccharomyces* yeasts are often used in combination with *Saccharomyces cerevisiae* to supplement alcohol production during the fermentation process (Lee et al., 2019; Lee & Park, 2020). Some aromatic compounds such as ethyl acetate and isoamyl acetate, which are prominently produced by non-*Saccharomyces* yeasts such as *Wickerhamomyces anomalus* and *Hanseniaspora uvarum*, serve as quality indicators for pleasant fruity or banana-like aromas when present in appropriate concentrations. Therefore, these compounds are recognized as superior aroma quality markers in alcoholic beverage research (Fan et al., 2019; Lee et al., 2019; Moreira et al., 2011). Similar to whiskies and brandies, the flavor of distilled *soju* is also intimately linked to the various volatile aromatic compounds from the head, heart, and tail fractions acquired during the distillation process, which is influenced by the volatility of these compounds produced during the fermentation (Kelly et al., 2023). In turn, volatility is influenced by factors such as boiling points, ethanol concentration, polarity, and vapor pressure during distillation, in addition to hydrogen bonds and carbon length variations, which influence the boiling points of aromatic compounds (Gopinath et al., 2015; Kelly et al., 2023). Many aromatic compounds with low boiling points, especially shorter-chain esters of 8 carbons and less, are collected in the heart fraction. Among these compounds, ethyl hexanoate and ethyl octanoate exhibit fruity aromas (Kelly et al., 2023; Stewart, 2017; Waymark & Hill, 2021). Similar to alcohol components, volatile aromatic compounds with lower boiling points are presumably concentrated during the main distillation recovery process of distilled *soju*, thereby enhancing their concentrations and influencing the quality of distilled *soju*. While non-*Saccharomyces* yeasts are known to generate aromatic compounds and elevate the levels of low-molecular-weight aromatic compounds during distillation, no previous studies have explored their effect on the quality of distilled *soju*.

In this study, we conducted co-fermentation of non-*Saccharomyces* yeasts and *S. cerevisiae* to induce the production of various volatile aromatic compounds during fermentation, thereby enhancing *takju* quality. Afterward, the components present in *takju* were concentrated during the distillation process and the effects of co-fermentation on the quality of distilled *soju* were evaluated.

## 2 Materials and methods

### 2.1 Strains and materials

*S. cerevisiae* NY-21 (KACC 93314P) previously isolated from *nuruk*, a traditional Korean fermentation starter used to make distilled *soju*, was used for *takju* fermentation (Park, Choi, Park, Lee, and Lee, 2021A). *W. anomalus* SJ20 (KACC 931297P) and *W. anomalus* JK04 (KACC 93193P) were previously isolated from persimmon and *nuruk*, respectively (Kim et al., 2019; Wahyono et al., 2016), and were used for co-fermentation to improve the quality of *takju*. For the production of *takju*, rice harvested in 2022 was purchased from DG Farm (Daegu, Korea). Traditional *nuruk* with an enzyme activity of 300 SP (saccharification power) or higher was purchased from Songhak Gokja Co., Ltd. (Gwangju, Korea). Here, SP was defined as the amount of enzyme that yields 1 mg of glucose per gram of sample per hour at 40 °C (Xi et al., 2023). The water used in this study was obtained from Hitejinro Co., Ltd. (Sejong, Korea). *S. cerevisiae* KCCM 11215, an industrial strain commonly used for the production of *takju* and distilled *soju*, was obtained from the Korean Culture Center of Microorganisms and was used as the control.

### 2.2 Preparation of *takju* and distilled *soju*

The initial brew (mother brew) was prepared by inoculating 300 g of *ipguk* (koji) with *Aspergillus oryzae* OF5–20 (Kim et al., 2020)*,* to which 450 mL of water and 15 mL of yeast culture were added. The mixture was then cultured for 36 h at 24 °C. Each sample was inoculated with *W. anomalus* and *S. cerevisiae* NY-21 at a 9:1 (*v*/v) ratio to delay alcohol production by *S. cerevisiae* and allow the non-*Saccharomyce*s yeast to exert a greater influence on the quality of *takju* during the fermentation process. Additionally, *S. cerevisiae* NY-21 and *S. cerevisiae* KCCM 11215 were individually inoculated as controls. Next, *ipguk*, steamed rice, and water were incorporated into the main fermentation process. In the initial stage of fermentation, 1700 g of *ipguk* and 2550 mL of water were added to a 20 L fermentation vessel, followed by the addition of precultured first brew. The mixture was then allowed to ferment at 24 °C for 2 days. Upon confirming successful fermentation, 4000 g of steamed rice and 6000 mL of water were added, and fermentation continued for approximately 11 days in a fermentation room at 20 °C. Fermentation was deemed complete when carbon dioxide production decreased significantly, and the alcohol content reached its peak. Once fermentation was completed, the *takju* was filtered through a 40-mesh (0.4 mm hole) cloth and then centrifuged at 4973 ×*g* for 10 min at 4 °C to remove the solid residues and for further analysis of various characteristics and components.

To produce distilled *soju*, the centrifuged *takju* was vacuum distilled using a small stainless steel still (20 L, Daeyoung Co., Gwangju, Korea). The distillation process was conducted with indirect heating at 65 °C using a jacketed system, and a reduced vacuum pressure of 560 ± 20 mmHg was maintained using a vacuum pump. The cooling water temperature was maintained at 5 °C, and approximately 3% of the total volume was drawn off as the heads, while the hearts were collected until the alcohol content reached approximately 45%. The initial alcohol content after *soju* distillation ranged from 42.03% to 44.02%. To mitigate the impact of high alcohol content on the sensory qualities of distilled *soju* and to allow for an objective comparison of the flavor characteristics of each sample, the final alcohol content of the distilled *soju* samples was adjusted to 25% using distilled water. Subsequently, further component analysis and sensory evaluations were conducted following the filtration and refinement processes (Table 1).

**Table 1:** Physicochemical properties of *takju* co-fermented with *Saccharomyces cerevisiae* and *Wickerhamomyces anomalus* strains and ethanol contents of distilled *soju.*

	Strains			
	S11215^1)^	NY21	NY21 + SJ20	NY21 + JK04
*Takju*				
Soluble solid (°Brix)	8.3 ± 0.1^2)b3)^	9.9 ± 0.0^a^	9.8 ± 0.1^a^	9.9 ± 0.0^a^
Reducing sugar (%)	0.21 ± 0.01^d^	0.44 ± 0.02^a^	0.33 ± 0.01^c^	0.38 ± 0.02^b^
pH	3.61 ± 0.01^a^	3.52 ± 0.01^b^	3.42 ± 0.07^b^	3.50 ± 0.02^b^
Total acidity (%)	0.67 ± 0.01^c^	0.83 ± 0.01^b^	0.87 ± 0.02^a^	0.86 ± 0.01^a^
Alcohol (%)	16.2 ± 0.1^a^	16.4 ± 0.1^a^	16.2 ± 0.1^a^	16.2 ± 0.1^a^
Ethanol content of distilled *soju* (%)				
After distillation	42.03	44.02	43.86	44.00
After ethanol adjustment	25	25	25	25

1) S11215, *S. cerevisiae* KCCM 11215; NY21, *S. cerevisiae* NY-21; SJ20, *W. anomalus* SJ20; JK04, *W. anomalus* JK04.

2) All data are expressed as the mean ± standard deviation (*n* = 3).

3) a-d Different letters within the same row indicate statistically significant differences (*p* < 0.05).

### 2.3 Physicochemical properties of *takju*

The physicochemical properties of *takju* were analyzed using a *takju* supernatant obtained by centrifugation (3578 ×*g*, 10 min). Soluble solids were then measured using a refractometer (RA 250, ATAGO, Tokyo, Japan). The reducing sugar content was determined by colorimetric quantification using the DNS (3,5-dinitrosalicylic acid, Sigma-Aldrich Co., St. Louis, MO, USA) reagent (Park, Choi, Park, Lee, and Park, 2021B). To induce a reaction, 1 mL of DNS was added to 0.3 mL of sample at 95 °C for 5 min, followed by the addition of 7 mL of distilled water. The absorbance of the sample was then measured using a spectrophotometer (UV-1601, Shimadzu, Co., Kyoto, Japan) at 550 nm, and the reducing sugar content was calculated from a glucose standard curve. pH was measured using a pH meter (SevenEasy S20, Mettler-Toledo, OH, USA), and total acidity was determined by titration of the filtrates with 0.1 N NaOH (expressed as g/L of lactic acid) (Choi et al., 2023). The alcohol content was determined by taking 100 mL of the supernatant obtained after centrifugation and transferring it to a distillation flask. The remaining residue in the measuring flask was rinsed twice with 15 mL of distilled water, and the washings were added to the distillation flask. Subsequently, the mixture was distilled to obtain 70 mL of distillate. The alcohol content of the mixed distillate was measured using a hydrometer based on the specific gravity of the *takju* distillates (expressed as % [*v*/v]), and the temperature was corrected to 15 °C using the Gay-Russac alcoholometric table (Choi et al., 2023).

### 2.4 Volatile aromatic compounds of *takju* and distilled *soju*

Prior to distillation, the volatile aromatic compounds were analyzed using the supernatant obtained from the centrifuged *takju*. After distillation, the alcohol content of the distilled soju was adjusted to 25% to conduct a comparative analysis of the relative volatile aromatic compounds under different conditions. The volatile aromatic compounds were quantified using gas chromatography–mass spectrometry (7890A, Agilent Technologies Inc., Santa Clara, CA, USA) equipped with a flame ionization detector (Agilent Technologies Inc.) described by Lee et al. (2016). Separation was performed using a DB-WAX column (60 m × 250 μm × 0.25 mm; Foods 2023, 12, 3246 5 of 17 Waters, Milford, MA, USA), and the compounds were detected using a triple-axis Agilent 5975C Inert XL MSD detector. Helium was used as the carrier gas at a consistent flow rate of 1 mL/min. The chromatograph oven was initially held at 40 °C for 2 min, after which the temperature was increased to 220 °C at a 2 °C/min rate, then to 240 °C at a 20 °C/min rate, with a final hold at 240 °C for 5 min. Volatile aromatic compounds were extracted from distilled *soju* using a solid-phase microextraction (SPME) fiber (50/30 μm DVB/CAR/PDMS; Supelco, Bellefonte, PA, USA). The extractions were conducted in headspace (HS) mode with magnetic stirring. Afterward, 5 mL of the sample was transferred to an HS vial (20 mm, PTFE/silicon septum, magnetic cap), along with the addition of 1.25 g of NaCl to enhance the concentration of the volatile aromatic compounds in the HS by increasing the retention of the water-soluble components. Prior to extraction, the sample was agitated in a water bath at 35 °C for 20 min to reach equilibrium. The SPME fiber was then inserted into the vial and allowed to react with its contents for 40 min at 30 °C. Commercial standards were quantified using the standard mixtures provided by MetaSci (Toronto, ON, Canada), with purities of at least 99.9%. The volatile aromatic compounds in the samples were identified by comparing their gas chromatograph retention times and mass spectra to the reference spectral data from the Wiley Registry 12th Edition/NIST 2020 Mass Spectral Library (John Wiley and Sons, Inc. Hoboken, NJ). The quantity of each compound in the *takju* and distilled *soju* samples was calculated based on the areas of the peaks identified using the chemical standards.

### 2.5 Sensory evaluation

To assess the influence of co-fermentation with *S. cerevisiae* and *W. anomalus* on the quality of the distilled *soju*, a sensory evaluation was performed with a panel of 20 individuals from the School of Food Science and Biotechnology at Kyungpook National University, Korea. These panelists were appropriately trained, experienced, and sensitive to taste discrimination. All participants provided consent to participate in the sensory evaluation and agreed to the use of their information. The sensory evaluation consisted of a seven-point scale to assess color, odor, sweetness, sourness, and overall preference. Prior to conducting the sensory evaluation, approval was obtained from the Institutional Review Board of Kyungpook National University (approval number 2022–0365).

### 2.6 Statistical analysis

All experiments were conducted at least in triplicate. The data are presented as mean values with standard deviation and were analyzed using the Statistical Package for the Social Sciences (SPSS, v. 12.0 for Windows). Pair-wise and multiple comparisons were respectively conducted via Student's *t*-test and one-way analysis of variance (ANOVA), followed by Duncan's multiple range tests. *P-values* < 0.05 were considered statistically significant.

## 3 Results and discussion

### 3.1 Physicochemical properties of *takju*

The physicochemical properties of *takju* fermented through single- or co-fermentation are presented in Table 1. Compared to the control *takju* fermented with the commercial yeast *S. cerevisiae* KCCM 11215, *takju* single-fermented with *S. cerevisiae* NY-21 or co-fermented with *S. cerevisiae* NY-21 and *W. anomalus* strains exhibited higher final soluble solid levels, reducing sugar content, and total acidity. The pH of *takju* single-fermented with *S. cerevisiae* NY-21 or co-fermented with *S. cerevisiae* NY-21 and *W. anomalus* strains was lower than that of the control *takju*. However, no significant differences in alcohol content were observed among any of the examined *takju* samples. The *takju* co-fermented with *S. cerevisiae* NY-21 and *W. anomalus* strains displayed slightly lower pH and higher total acidity compared to *takju* single-fermented with *S. cerevisiae* NY-21. After distillation, the alcohol content ranged from 42.03% to 44.02%. To ensure consistency in component analysis and sensory evaluation across all *takju* samples, distilled water was added to adjust the final alcohol content to 25%.

*S. cerevisiae* NY-21 was previously isolated for the production of distilled *soju* and has been shown to produce higher levels of alcohol and organic acids such as tartaric acid, malic acid, and succinic acid after fermentation compared with commercial yeasts such as La Parisienne, SafSpirit HG-1, and EC-1118 (Park, Choi, Park, Lee, and Lee, 2021A). Our findings suggest that *S. cerevisiae* NY-21 plays a role in lowering pH and increasing total acidity compared to *S. cerevisiae* KCCM 11215. *W. anomalus* is known to use malic acid as a sole carbon source. However, this ability is suppressed in the presence of sugars (Corte-Real & Leao, 1990). In a study by Satora et al. (2014), co-fermentation of unpasteurized apple cider with *S. cerevisiae* JR and *W. anomalus* strains (CBS 1982 and CBS 5759) led to increased volatile acidity, titratable acidity, and higher malic acid content compared to single fermentation with *S. cerevisiae*. This suggests that the presence of residual malic acid during co-fermentation with *W. anomalus* strains contributes to the higher total acidity observed in *takju* co-fermented with *S. cerevisiae* NY-21 compared to *takju* single-fermented with *S. cerevisiae* NY-21.

### 3.2 Changes in the volatile aromatic compounds of *takju* before and after distillation

The contents of volatile aromatic compounds in *takju* (before distillation) and distilled *soju* (after distillation) are presented in Table 2, Table 3, respectively. In *takju* fermented with *S. cerevisiae* NY-21, the contents of ethyl acetate, methyl salicylate, ethyl dodecanoate, ethyl tetradecanoate, ethyl hexadecanoate, and ethyl linoleate were significantly higher than those of the control *takju* fermented with *S. cerevisiae* KCCM 11215. Slight increases in ethyl decanoate and ethyl oleate were also observed. Although the levels of aldehyde and higher alcohol groups tended to decrease in *takju* fermented with *S. cerevisiae* NY-21 compared with those of *takju* fermented with *S. cerevisiae* KCCM 11215, there was a significant overall increase in esters, indicating that *S. cerevisiae* NY-21 enhanced the qualities of *takju* compared with *S. cerevisiae* KCCM 11215. Moreover, compared with *takju* fermented exclusively with *S. cerevisiae* NY-21, *takju* co-fermented with *S. cerevisiae* NY-21 and *W. anomalus* strains exhibited a significant increase in ethyl acetate, isoamyl acetate, ethyl octanoate, ethyl decanoate, and ethyl dodecanoate contents, which impart fruity aromas. Small increases in 2-phenylethyl acetate and ethyl tetradecanoate levels were also observed. Similarly, slight increases in the overall levels of esters were also observed in *takju* co-fermented with *S. cerevisiae* NY-21 and *W. anomalus* strains compared with *takju* single-fermented with *S. cerevisiae* NY-21. Furthermore, higher levels of aldehydes and alcohol contents were observed in *takju* co-fermented with *S. cerevisiae* NY-21 and *W. anomalus* strains compared to *takju* single-fermented with *S. cerevisiae* NY-21. These findings suggest that co-fermentation with *S. cerevisiae* NY-21 and *W. anomalus* contributes to the production of a wider range of volatile aromatic compounds compared with single-fermentation with *S. cerevisiae* NY-21. After distillation, the difference in the levels of volatile aromatic compounds between distilled *soju* produced through co-fermentation with *S. cerevisiae* NY-21 and *W. anomalus* strains and distilled *soju* produced through single fermentation with *S. cerevisiae* NY-21 was significantly greater than that of *takju* before distillation. Particularly, the overall levels of esters were markedly decreased, whereas the overall levels of higher alcohols were increased (Table 3). However, despite this decrease in the overall levels of esters in distilled *soju*, distilled *soju* produced by co-fermentation with *S. cerevisiae* NY-21 and *W. anomalus* strains exhibited significantly higher levels of esters, higher alcohols, and total volatile aromatic compounds compared with distilled *soju* produced by single fermentation with *S. cerevisiae* NY-21.

**Table 2:** Volatile aromatic compound contents of *takju* (before distillation) co-fermented with *Saccharomyces cerevisiae* and *Wickerhamomyces anomalus* strains.

Compounds	Odor description^1)^	Volatile aromatic compound (mg/L)			
		S11215^2)^	NY21	NY21 + SJ20	NY21 + JK04
Esters					
Ethyl acetate	Fruity, sweet	87.33 ± 5.89^3)d4)^	150.27 ± 14.66^c^	188.23 ± 16.87^b^	226.52 ± 23.73^a^
Isoamyl acetate	Banana, pear	148.55 ± 13.67^a^	84.58 ± 9.82^c^	106.88 ± 10.42^b^	124.23 ± 12.08^b^
Ethyl hexanoate	Fruity, apple, banana	7.27 ± 0.64^b^	6.57 ± 0.72^b^	10.57 ± 1.12^a^	8.17 ± 1.08^b^
Ethyl octanoate	Pineapple, pear	17.35 ± 1.39^b^	10.37 ± 0.86^c^	25.06 ± 2.31^a^	25.67 ± 2.42^a^
Ethyl decanoate	Fatty acids, fruity, apple, solvent	39.40 ± 3.58^b^	43.96 ± 4.15^b^	63.92 ± 6.79^a^	73.32 ± 6.98^a^
Methyl salicylate	Wintergreen pepper, mint	185.65 ± 15.50^b^	252.26 ± 23.36^a^	169.34 ± 15.88^b^	175.10 ± 16.32^b^
2-Phenylethyl acetate	Fruity, flower	856.88 ± 69.07^a^	249.80 ± 27.61^b^	274.72 ± 25.40^b^	267.86 ± 24.60^b^
Ethyl dodecanoate	Oily, fatty, fruity	22.56 ± 2.04^d^	108.02 ± 11.13^c^	136.98 ± 12.37^b^	160.40 ± 16.32^a^
Ethyl tetradecanoate		150.66 ± 16.83^b^	1164.84 ± 130.61^a^	1378.57 ± 128.33^a^	1361.84 ± 132.17^a^
Ethyl hexadecanoate		3774.77 ± 254.83^b^	8912.23 ± 749.85^a^	9490.96 ± 905.62^a^	8900.68 ± 852.41^a^
Ethyl octadecanoate		197.25 ± 16.63^a^	133.60 ± 14.30^b^	112.37 ± 12.21^b^	111.08 ± 10.47^b^
Ethyl oleate		923.66 ± 90.71^a^	924.04 ± 91.80^a^	843.53 ± 86.57^a^	820.03 ± 81.35^a^
Ethyl linoleate		809.68 ± 74.88^b^	1138.51 ± 110.49^a^	1120.17 ± 109.05^a^	1092.99 ± 105.83^a^
Subtotal		7221.02 ± 565.66^b^	13,179.06 ± 1189.36^a^	13,921.30 ± 1332.94^a^	13,347.89 ± 1285.76^a^
Aldehyde					
Diethyl acetal	Pungent, green, woody solvent	ND^5)^	ND	ND	ND
Furfural	Almond	11.49 ± 0.98^a^	8.86 ± 0.79^b^	6.64 ± 0.60^c^	7.30 ± 0.72^c^
2-Methylbenzaldehyde		31.25 ± 3.30^b^	1.91 ± 0.30^c^	58.03 ± 5.33^a^	60.82 ± 6.55^a^
4-propyl benzaldehyde		2.73 ± 0.25^b^	14.85 ± 1.14^a^	4.13 ± 0.31^b^	2.87 ± 0.30^b^
Subtotal		45.47 ± 4.53^b^	25.62 ± 2.23^c^	68.81 ± 6.24^a^	70.98 ± 7.57^a^
Higher alcohol					
Isobutanol	Alcohol, solvent, green, bitter	159.71 ± 13.68^b^	146.26 ± 14.30^b^	209.45 ± 19.76^a^	241.37 ± 22.41^a^
Isoamyl alcohol	Solvent, sweet, nail polish	803.65 ± 81.26^b^	740.04 ± 72.68^b^	949.43 ± 92.58^b^	1134.81 ± 114.37^a^
Dimethylsilanediol		10.97 ± 1.10^b^	11.07 ± 1.09^b^	13.47 ± 1.29^b^	30.17 ± 2.86^a^
Phenylethyl alcohol	Rose, honey	1964.42 ± 206.09^a^	1071.17 ± 114.01^b^	1087.67 ± 105.02^b^	1145.08 ± 108.56^b^
Subtotal		2938.75 ± 302.13^a^	1968.54 ± 202.08^c^	2260.02 ± 218.65^bc^	2551.43 ± 248.20^ab^
Total		10,205.24 ± 872.32^b^	15,173.23 ± 1393.67^a^	16,250.13 ± 1557.83^a^	15,970.30 ± 1541.53^a^

1) Choi et al. (2020).

2) S11215, *S. cerevisiae* KCCM 11215; NY21, *S. cerevisiae* NY-21; SJ20, *W. anomalus* SJ20; JK04, *W. anomalus* JK04.

3) All data are expressed as the mean ± standard deviation (*n* = 3).

4) a-d Different letters within the same row indicate statistically significant differences (*p* < 0.05).

5) ND, not detected.

**Table 3:** Volatile aromatic compound contents of distilled *soju* (after distillation) prepared with *takju* co-fermented with *Saccharomyces cerevisiae* and *Wickerhamomyces anomalus* strains.

Compounds	Odor description^1)^	Boiling point (°C)	Volatile aromatic compound (mg/L)			
			S11215^2)^	NY21	NY21 + SJ20	NY21 + JK04
Esters						
Ethyl acetate	Fruity, sweet	77.1	325.43 ± 28.59^3)b4)^	307.40 ± 30.20^b^	583.73 ± 56.31^a^	504.55 ± 51.25^a^
Isoamyl acetate	Banana, pear	142.0	192.00 ± 20.33^a^	54.92 ± 6.13^c^	150.37 ± 14.64^b^	133.03 ± 12.26^b^
Ethyl hexanoate	Fruity, apple, banana	168.0	1100.67 ± 105.94^c^	41.32 ± 4.58^d^	2046.44 ± 185.13^a^	1530.47 ± 146.60^b^
Ethyl octanoate	Pineapple, pear	208.0	15.29 ± 1.36^b^	9.99 ± 1.03^c^	19.58 ± 1.84^a^	11.94 ± 1.09^c^
Ethyl decanoate	Fatty acids, fruity, apple, solvent	245.0	26.18 ± 3.01^a^	13.70 ± 1.12^b^	11.04 ± 1.09^b^	12.02 ± 1.16^b^
Methyl salicylate	Wintergreen pepper, mint	220.0	16.61 ± 1.58^a^	11.06 ± 1.05^b^	11.62 ± 1.14^b^	12.15 ± 1.28^b^
2-Phenylethyl acetate	Fruity, flower	232.6	309.50 ± 31.62^a^	89.83 ± 9.17^b^	120.87 ± 11.67^b^	92.90 ± 9.12^b^
Ethyl dodecanoate	Oily, fatty, fruity	269.0	39.95 ± 4.01^a^	38.46 ± 3.76^a^	17.24 ± 1.56^c^	29.67 ± 3.13^b^
Ethyl tetradecanoate		295.0	105.79 ± 11.27^c^	272.98 ± 25.64^a^	119.59 ± 13.03^c^	230.62 ± 22.72^b^
Ethyl hexadecanoate		377.0	878.49 ± 85.63^c^	2803.08 ± 272.59^a^	2005.52 ± 206.11^b^	2800.01 ± 265.74^a^
Ethyl octadecanoate			39.16 ± 3.54^c^	171.67 ± 16.83^a^	125.64 ± 11.27^b^	137.02 ± 14.31^b^
Ethyl oleate			125.63 ± 10.71^b^	650.05 ± 62.71^a^	560.33 ± 53.65^a^	569.68 ± 55.28^a^
Ethyl linoleate			36.35 ± 2.27^b^	354.21 ± 34.17^a^	360.57 ± 34.05^a^	377.14 ± 32.93^a^
Subtotal			3214.02 ± 309.86^c^	4832.34 ± 468.98^b^	6149.33 ± 591.49^a^	6449.83 ± 616.87^a^
Aldehyde						
Diethyl acetal	Pungent, green, woody solvent	102.0	209.79 ± 18.58^a^	195.39 ± 19.65^a^	14.28 ± 1.34^c^	135.48 ± 12.65^b^
Furfural	Almond	162.0	ND^5)^	ND	ND	ND
2-Methylbenzaldehyde		200.0	ND	ND	ND	ND
4-propyl benzaldehyde			ND	ND	ND	ND
Subtotal			209.79 ± 18.58^a^	195.39 ± 19.65^a^	14.28 ± 1.34^c^	135.48 ± 12.65^b^
Higher alcohol						
Isobutanol	Alcohol, solvent, green, bitter	108.0	2997.39 ± 301.63^a^	1946.23 ± 192.77^b^	2512.23 ± 242.41^a^	3089.61 ± 305.58^a^
Isoamyl alcohol	Solvent, sweet, nail polish	131.0	5473.12 ± 516.72^ab^	4773.35 ± 438.75^b^	6416.40 ± 627.83^a^	5771.42 ± 529.70^ab^
Dimethylsilanediol		100.0	39.73 ± 3.67^b^	47.16 ± 4.62^b^	78.10 ± 7.36^a^	81.88 ± 7.99^a^
Phenylethyl alcohol	Rose, honey	225.0	553.94 ± 52.32^a^	243.38 ± 22.63^c^	329.38 ± 31.66^b^	294.58 ± 28.36^bc^
Subtotal			9064.19 ± 874.34^a^	7010.12 ± 658.77^b^	9336.12 ± 909.26^a^	9237.49 ± 871.63^a^
Total			12,488.00 ± 1202.78^b^	12,037.85 ± 1147.40^b^	15,499.72 ± 1502.09^a^	15,822.80 ± 1501.15^a^

1) Choi et al. (2020).

2) S11215, *S. cerevisiae* KCCM 11215; NY21, *S. cerevisiae* NY-21; SJ20, *W. anomalus* SJ20; JK04, *W. anomalus* JK04.

3) All data are expressed as the mean ± standard deviation (*n* = 3).

4) a-d Different letters within the same row indicate statistically significant differences (*p* < 0.05).

5) ND, not detected.

**Fig. 1:**
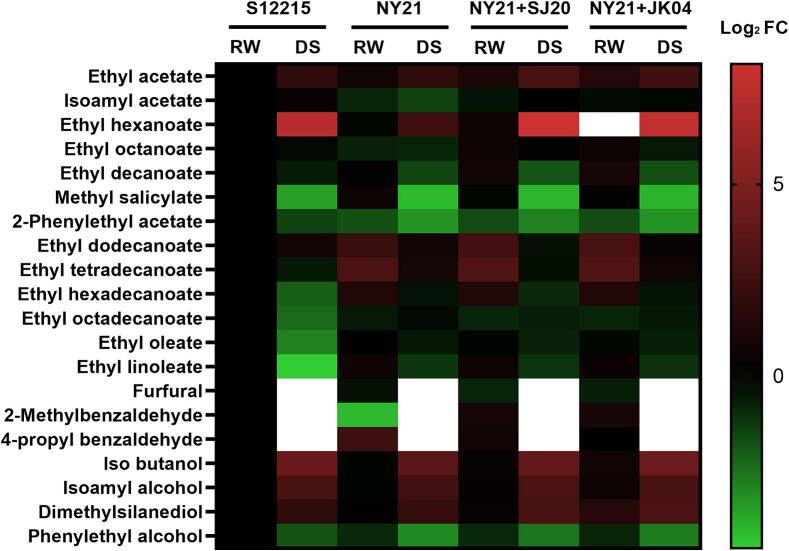
Heatmap showing the relative changes in volatile aromatic compounds between *takju* (RW) and distilled *soju* (DS) obtained via co-fermentation with *Saccharomyces cerevisiae* and *Wickerhamomyces anomalus* strains. Log_2_ fold change values were compared between co-fermented *takju* and *takju* single-fermented with *S. cerevisiae* S12215. The white boxes indicate undetected compounds. Abbreviations: S11215, *S. cerevisiae* KCCM 11215; NY21, *S. cerevisiae* NY-21; SJ20, *W. anomalus* SJ20; JK04, *W. anomalus* JK04.

When comparing the levels of volatile aromatic compounds between *takju* (pre-distillation) and distilled *soju* (post-distillation) and converting the changes into Log2 fold change (FC) values (Fig. 1), we observed that the substances with boiling points below 200 °C were markedly concentrated in the distilled *soju* (excluding furfural). In contrast, compounds with higher boiling points tended to remain in the tail without being collected into the final distilled *soju*. Specifically, we observed an increase in the levels of ethyl acetate, isoamyl acetate, ethyl hexanoate, diethyl acetal, isobutanol, isoamyl alcohol, and dimethyl silanediol, with ethyl hexanoate, diethyl acetal, isobutanol, and isoamyl alcohol exhibiting a significant increase. These compounds were identified as significant volatile aromatic compounds that have a substantial impact on the quality of distilled *soju*. Conversely, methyl salicylate, 2-phenylethyl acetate, ethyl dodecanoate, ethyl tetradecanoate, ethyl hexadecanoate, ethyl linoleate, and phenylethyl alcohol contents, which are considered major aromatic compounds in *takju*, were significantly decreased, indicating a distinct change in aromatic compound patterns between pre-distillation (*takju*) and post-distillation (distilled *soju*).

Numerous studies have demonstrated that non-*Saccharomyces* yeasts can enhance the flavor of fermented foods such as wine, apple cider, persimmon wine, rice wine, and soy sauce by enhancing the production of various aromatic compounds during fermentation (Chen et al., 2021; Gao et al., 2023; Kim et al., 2019; Lee et al., 2019; Wee et al., 2018; Yan et al., 2019). Among them, *W. anomalus* effectively produces ethyl acetate, isoamyl acetate, and other compounds during the early stages of fermentation, and it is commonly used in co-fermentation as a non-*Saccharomyces* yeast (Satora et al., 2014; Yao et al., 2023). When co-fermented with *S. cerevisiae* Y3401 and *W. anomalus* Y3604, *baijiu*, a Chinese distilled spirit, exhibited a significant increase in ethyl acetate content, a key aromatic compound that influences the quality of *baijiu* (Fan et al., 2019). In this study, two strains of *W. anomalus* were combined with *S. cerevisiae* to ferment *takju*, and the contents of the volatile aromatic compounds, such as ethyl acetate, isoamyl acetate, ethyl octanoate, ethyl decanoate, ethyl dodecanoate, 2-methylbenzaldehyde, and isobutanol, were significantly increased. Additionally, there was a slight increase in the content of ethyl hexanoate, 2-phenylethyl acetate, ethyl tetradecanoate, isoamyl alcohol, dimethylsilanediol, and phenylethyl alcohol, suggesting that *W. anomalus* imparted floral, fruity, and sweet aromas during fermentation, thereby enhancing the flavor characteristics of *takju*. Compared with the control *takju* single-fermented with *S. cerevisiae* KCCM 11215, *takju* single-fermented with *S. cerevisiae* NY-21 exhibited an increase in total ester content but a decrease in the levels of total aldehydes and higher alcohols. Co-fermentation with *S. cerevisiae* NY-21 and *W. anomalus* results in higher levels of volatile aromatic compounds such as isoamyl acetate, ethyl octanoate, 2-phenylethyl acetate, 2-methylbenzaldehyde, isobutanol, isoamyl alcohol, and phenylethyl alcohol. In contrast, *takju* single-fermented with *S. cerevisiae* NY-21, exhibited lower levels of the aforementioned aromatic compounds, demonstrating that co-fermentation with *S. cerevisiae* NY-21 and *W. anomalus* strains improves the quality of *takju*, and presumably also influencing the quality of distilled *soju*. During the distillation process, these volatile aromatic compounds separate at different stages depending on their boiling point, their solubility in the alcohol and water mixture, and the type of distillation equipment used (Balcerek et al., 2017). In a typical distillation process, alcohol is separated into three fractions, with the head fraction containing aliphatic aldehydes such as acetaldehyde and esters (e.g., ethyl acetate, isoamyl acetate, and methyl acetate) with low boiling points and high solubility in ethanol (Léauté, 1990). According to Balcerek et al. (2017), the head fractions of plum brandy (initial 10% of the alcohol volume, 100% *v*/v present in the raw distillate) mainly contain aliphatic aldehydes, acetals, and esters, as well as higher alcohols (1-propanol, 2-methyl-1-propanol, 1-butanol, 2-methyl-1-butanol, and 3-methyl-1-butanol). Additionally, the authors reported that the tail fractions contained relatively high concentrations of furfural, 1-hexanol, benzyl alcohol, 2-phenylethanol, and ethyl carbamate. In our study, 3% of the spirit's volume was removed as the head fraction, and it appeared that some ethyl acetate and isoamyl acetate were collected during the early part of the heart fraction. Moreover, the contents of ethyl hexanoate, isobutanol, isoamyl alcohol, and dimethylsilanediol, considered part of the heart fraction, were significantly higher in the distilled *soju*. Zhao et al. (2022) reported that isoamyl alcohol, phenylethanol, ethyl palmitate, isobutyl acetate, and ethyl myristate are major aromatic compounds in *huangjiu*, a type of Chinese rice wine. Isoamyl alcohol contributes significantly to the flavor profile of rice-based fermented beverages such as *baijiu*, *awamori*, and *kome*-*sochou*, with isobutanol, phenylethyl alcohol, and ethyl caproate (ethyl hexanoate) also being recognized as key flavor compounds in *baijiu*, as reported by Yin et al. (2020). Goss et al. (1999) reported that primary fatty acid ethyl esters in Scotch whiskey were of medium chain length (C10), whereas esters with C14 or more carbons only occurred in trace amounts due to their reduced solubility with longer carbon chains. In this study, we observed significant increases in the content of ethyl hexanoate, isobutanol, and isoamyl alcohol after distillation, highlighting their contribution as major aromatic compounds in distilled *soju*. Collectively, our findings indicated that co-fermentation with *S. cerevisiae* and *W. anomalus* increases the levels of these compounds, thereby enhancing the quality of distilled *soju*.

### 3.3 Sensory evaluation of distilled *soju*

Table 4 represents the results of the sensory evaluation conducted by 20 panelists from the School of Food Science and Biotechnology, Kyungpook National University. Sensory evaluation was conducted to investigate the impact of co-fermentation with *S. cerevisiae* NY-21 and *W. anomalus* strains on the sensory quality of distilled *soju*. According to the evaluation, distilled *soju* produced through co-fermentation with *S. cerevisiae* NY-21 and *W. anomalus* strains received superior scores in the odor evaluation. This result is likely attributed to the higher content of low-molecular-weight esters and higher alcohols among the volatile aromatic compounds in co-fermented distilled *soju*, which contribute to a more favorable aroma profile. Although the sweetness scores were lower in co-fermented distilled *soju* compared to those of distilled *soju* single-fermented with *S. cerevisiae* KCCM 11215 or *S. cerevisiae* NY-21, the overall preference scores indicated a higher preference for the distilled *soju* produced through co-fermentation with *S. cerevisiae* NY-21 and *W. anomalus* strains over the distilled *soju* produced through single fermentation with *S. cerevisiae* strains. This suggests that the inclusion of *W. anomalus* yeast in co-fermentation improves the overall quality and sensory appeal of distilled *soju*.

**Table 4:** Sensory evaluation of distilled *soju* prepared with *takju* co-fermented with *Saccharomyces cerevisiae* and *Wickerhamomyces anomalus* strains.

	S11215^1)^	NY21	NY21 + SJ20	NY21 + JK04
Color	4.4 ± 0.3^2)a3)^	4.4 ± 0.3^a^	4.3 ± 0.3^a^	4.5 ± 0.3^a^
Odor	3.4 ± 0.2^b^	5.5 ± 0.2^a^	5.9 ± 0.2^a^	5.7 ± 0.2^a^
Sweetness	4.0 ± 0.4^a^	3.5 ± 0.4^ab^	3.5 ± 0.4^b^	2.8 ± 0.3^ab^
Sourness	2.8 ± 0.3^a^	3.0 ± 0.4^a^	3.0 ± 0.3^a^	3.5 ± 0.4^a^
Overall preference	4.1 ± 0.3^b^	5.3 ± 0.2^a^	5.7 ± 0.4^a^	5.5 ± 0.3^a^

1) S11215, *S. cerevisiae* KCCM 11215; NY21, *S. cerevisiae* NY-21; SJ20, *W. anomalus* SJ20; JK04, *W. anomalus* JK04.

2) All data are expressed as the mean ± standard deviation (*n* = 20).

3) a-d Different letters within the same row indicate statistically significant differences (p < 0.05).

## 4 Conclusion

In this study, we explored the effects of co-fermentation with non-*Saccharomyces* yeast on the quality of distilled *soju*. Our findings revealed an increase in various volatile aromatic compounds in *takju* when subjected to co-fermentation with *W. anomalus*. Moreover, during the distillation process, certain compounds with boiling points below 200 °C were notably concentrated. Specifically, ethyl hexanoate, isobutanol, and isoamyl alcohol were identified as major aromatic compounds in distilled *soju* due to their substantial concentrations. However, it is important to note that the compounds concentrated during distillation can vary based on factors such as pressure, temperature, and the timing of the heart fraction collection. Nonetheless, our results indicate that co-fermentation with *W. anomalus* increased the levels of flavor-enhancing compounds, thereby significantly improving the overall quality of distilled *soju*.

## CRediT authorship contribution statement

**Kyu-Taek Choi:** Methodology, Conceptualization. **Yeong-Jun Kim:** Validation, Methodology, Investigation. **Jun-Su Choi:** Writing – review & editing, Validation, Data curation. **Sae-Byuk Lee:** Writing – review & editing, Supervision, Resources, Funding acquisition, Conceptualization.

## Declaration of competing interest

The authors declare that they have no known competing financial interests or personal relationships that could have appeared to influence the work reported in this paper.

## Data Availability

Data will be made available on request.
